# New Peptides Isolated from *Lyngbya* Species: A Review

**DOI:** 10.3390/md8061817

**Published:** 2010-06-09

**Authors:** Li Liu, Kathleen S. Rein

**Affiliations:** Department of Chemistry and Biochemistry, Florida International University, Miami, FL 33199, USA; E-Mail: lliu004@fiu.edu

**Keywords:** Lyngbya, cyanobacteria, peptide, secondary metabolites, bioactivity

## Abstract

Cyanobacteria of the genus *Lyngbya* have proven to be prodigious producers of secondary metabolites. Many of these compounds are bioactive and show potential for therapeutic use. This review covers peptides and hybrid polyketide-non-ribosomal peptides isolated from *Lyngbya* species. The structures and bioactivities of 50 *Lyngbya* peptides which were reported since 2007 are presented.

## 1. Introduction

Cyanobacteria, also called blue-green algae, are ancient aquatic and photosynthetic prokaryotes. The oldest known fossils of cyanobacteria come from Archaean rocks of Western Australia, dated at 3.5 billion years [1]. Over 300 nitrogen-containing secondary metabolites from marine cyanobacteria have been reported in the literature [2]. It is possible that the ability to produce a wide range of defensive secondary metabolites has contributed to the high degree biological adaptation observed for cyanobacteria [3–6]. These secondary metabolites often enable cyanobacteria to compete effectively in a variety of environments, and many have been presented as lead compounds for further drug development. For instance, the synthetic analog cryptophycin 52 (**1**), which progressed to Phase II clinical trials for the treatment of patients with platinum-resistant ovarian cancer, is based on the cryptophycin 1 (**2**) which was isolated from terrestrial cyanobacteria [7,8]. Phase II clinical trials of dolastatin 10 failed to show significant anticancer activity. However, *in vitro* studies of soblidotin (or TZT-1027, auristatin PE) (**4**) a synthetic analog of dolastatin 10 (**3**), showed promising results against human colon adenocarcinomas and has progressed to phase II clinical trials [9,10]. Synthadotin (or ILX-651) (**6**), derived from dolastatin 15 (**5**) showed promising results in phase II clinical trials of inoperable, locally advanced or metastatic melanoma [9,11].

**Figure:**
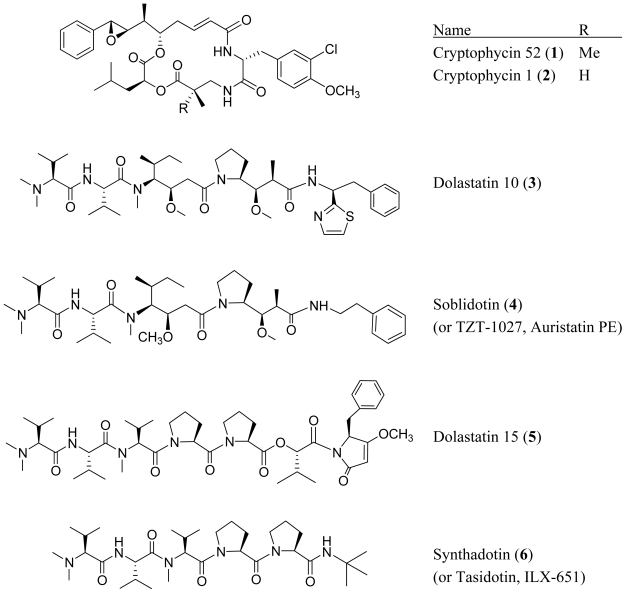


Cyanobacteria of toxicological or pharmacological significance include the genera *Anabaena*, *Oscillatoria*, *Microcystis*, *Nodularia*, *Cylindrospermopsis* and *Lyngbya. Lyngbya* sp. and *Microcystis* sp. are easily collected and cultured in the laboratory so that the isolation of compounds in the mg range is possible [12]. *Lyngbya* is a common and accessible genus of cyanobacteria, which is distributed worldwide throughout tropical and subtropical regions. The unbranched filaments of *Lyngbya* are cylindrical and usually wider than 6 μm. The straight, slightly wavy, or rarely coiled *Lyngbya* filaments usually form large, layered, leathery mats of varied thickness, and then form large benthic or surface blooms in freshwater and sea water. There are an increasing number of *Lyngbya* species which have been found to produce an impressive array of structurally varied compounds with diverse biological activities [4]. To date, the most important species of genus *Lyngbya* in terms of secondary metabolite production are *L. majuscula*, *L. martensiana*, *L. aestuarii* and *L. wollei*. Peptides or peptide containing substructures comprise a major group of cyanobacterial secondary metabolites. Many of these compounds were reviewed in 2006 [2]. This review covers new peptides reported from *Lyngbya* species after 2006, with an emphasis on their structures and biological activities. However, conflicting taxonomic identification of cyanobacteria, including the genus *Lyngbya*, is common [13]. In most instances, the taxonomic identification was made on the basis of morphological features. Less frequently, identification is made on the basis of rRNA gene sequences. Even when sequence data is available, existing databases are often not adequate for definitive identification even at the genus level. The majority of biological activities reported for the compounds fall into two major categories: cytotoxicity or protease inhibition.

## 2. Acyclic Peptides

Six analogs of dragonamide A (**7**) [14], including carmabin A (**8**), dragomabin (**9**) and dragonamide B (**10**) [15], dragonamides C–D (**11**–**12**) [16] and E (**13**) [17], were isolated from the marine cyanobacteria *Lyngbya majuscula* and *Lyngbya polychroa*. This series possesses an 8 or 10-carbon long terminal alkynamide. To the best of our knowledge, carmabin A (**8**), dragomabin (**9**), and dragonamide A (**7**) were the only *Lyngbya* metabolites showing good antimalarial activity (IC_50_ = 4.3, 6.0, and 7.7 μM, respectively). Dragonamides A (**7**) and E (**13**) were also antileishmanial against *Leishmania donovani* with IC_50_ values of 6.5 and 5.1 μM. However, the nonaromatic analog, dragonamide B (**10**), was inactive against malaria or leishmaniasis. The lack of activity for dragonamide B (**10)** suggests that an aromatic amino acid at the carboxy terminus is necessary for antiparasitic activity in this series. Carmabin A (**8**) was more cytotoxic to Vero cells (IC_50_ = 9.8 μM) than dragomabin (**5**) (IC_50_ = 182.3 μM) or dragonamide A (**7**) (IC_50_ = 67.8 μM). Thus, dragomabin (**9**) possesses the best differential toxicity between parasite and mammalian cells. It appears that the longer and more branched alkynamide chain of carmabin A (**8**) leads to the increase in cytotoxicity over that of dragomabin (**9**). Dragonamides C–D (**11**–**12**) showed weak activity in cancer cell viability assays, with the 50% growth inhibition (GI_50_) values of 56 and 59 μM against U2OS osteosarcoma cells, 22 and 32 μM against HT29 colon adenocarcinoma cells, and 49 and 51 μM against IMR-32 neuroblastoma cells, respectively. These data are similar to cytotoxicity data reported for dragonamides A (**7**) and B (**10**) against other cell lines. The antiparasitic activity of dragonamides C–D (**11**–**12**) was not reported.

Screening of marine cyanobacteria from the Caribbean coast of Panama led to the identification of two antileishmanial lipopeptides, almiramides B–C (**15**–**16**) and their non-active analog, almiramide A (**14**) from *Lyngbya majuscula* [18]. Compared with the most closely related secondary metabolite dragonamide A (**7**), almiramides B–C (**15**–**16**) with an extra Ala residue, no methyl group on Val1 and the opposite configuration of the α-carbon of the lipophilic side chain, showed better antileishmanial potencies (IC_50_ = 2.4, and 1.9 μM, respectively), but no antimalarial activity up to 13.5 μM. The lack of antileishmanial activity for almiramide A (**14**) indicated that an unsaturated terminus on the lipophilic side chain played a critical role for antileishmanial activity in dragonamides and almiramides. Screening of a synthetic library of almiramide analogs with various modifications at the C- or N-terminus afforded several compounds with similar antileishmanial activity but improved selectivity.

**Table:** 

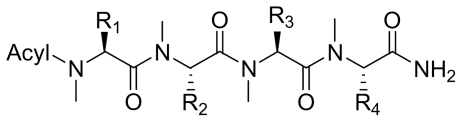					
Name	R_1_	R_2_	R_3_	R_4_	Acyl
Dragonamide A (**7**)	*i*-Pr	*i*-Pr	*i*-Pr	PhCH_2_-	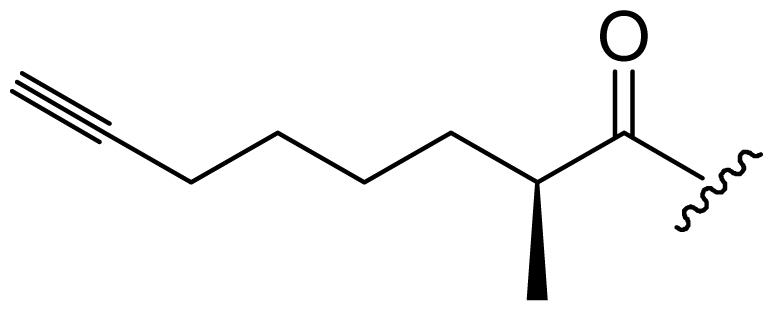
Carmabin A (**8**)	Bn	Me	Me	4-MeO-PhCH_2_-	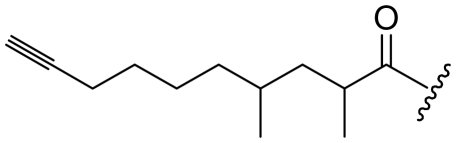
Dragomabin (**9**)	Bn	Me	Me	4-MeO-PhCH_2_-	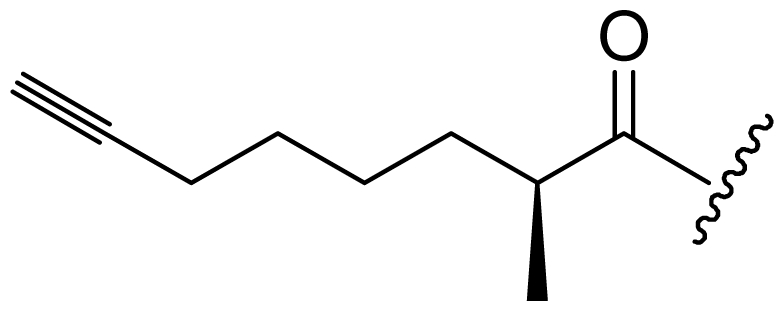
Dragonamide B (**10**)	*i*-Pr	*i*-Pr	*i*-Pr	*i*-Pr	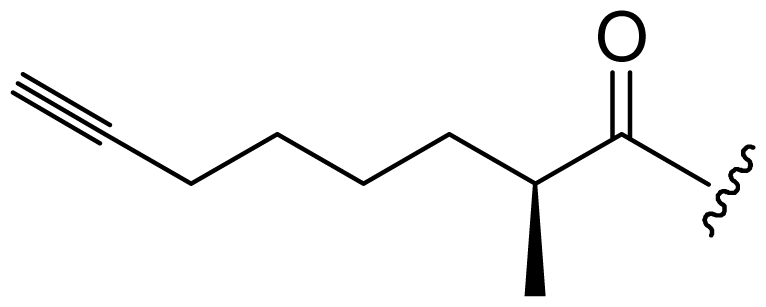
Dragonamide C (**11**)	*i*-Pr	*i*-Pr	*i*-Pr	*i*-Pr	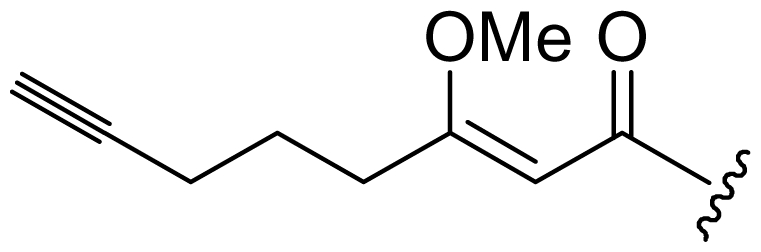
Dragonamide D (**12**)	*i*-Pr	*i*-Pr	*i*-Pr	*i*-Pr	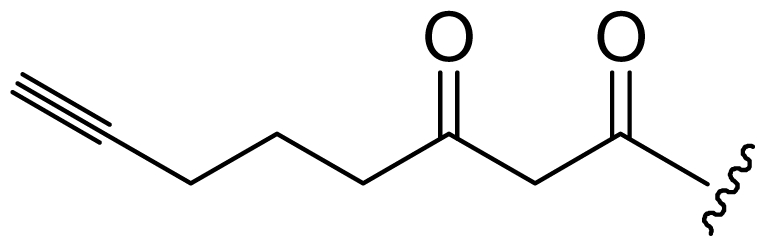
Dragonamide E (**13**)	*i*-Pr	*i*-Pr	*i*-Pr	PhCH_2_-	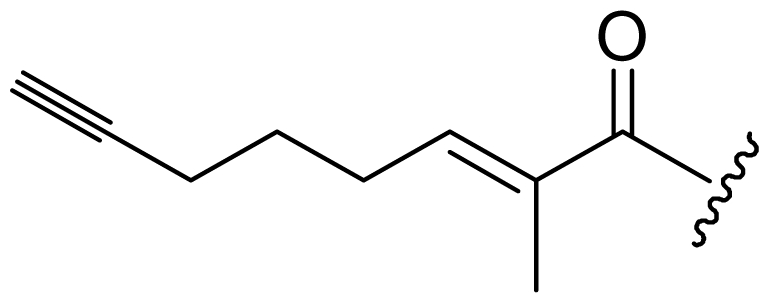

**Figure:**
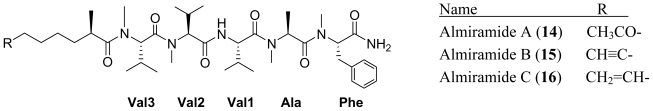


Efforts toward finding marine cyanobacterial metabolites with antitumor activity led to the isolation of bisebromoamide (**17**) from a *Lyngbya* sp. harvested in Okinawa Prefecture [19]. Bisebromoamide (**17**) is featured by four unusual structural units, the 2-substituted thiazoline-4-methyl-4-carboxylic acid unit fused to a methyl-proline, 2-(1-oxopropyl) pyrrolidine (Opp) residue, *N*-methyl-bromo-tyrosine and *N*-pivalamide moiety. Prior to the isolation of bisebromoamide (**17**), the Opp unit in had not been observed in any natural product. Bisebromoamide (**17**) showed cytotoxicity against HeLa S3 cells (IC_50_ = 0.04 μg/mL) and a panel of 39 human cancer cell lines (termed JFCR39) (the average GI_50_ = 40 nM). At 10 to 0.1 μM, bisebromoamide (**17**) selectively inhibited the phosphorylation of ERK (extracellular signal regulated protein kinase) in NRK (normal rat kidney) cells by PDGF (platelet-derived growth factor)-stimulation. However PKB (protein kinase B), PKD (protein kinase D), PLCγ1 (phospholipase Cγ1), or S6 ribosomal protein was not affected by bisebromoamide (**17**) at the same concentration range. Bisebromoamide (**17**) did not affect tubulin acylation as other tubulin modulators. It is possible that bisebromoamide (**17**) targets the ERK signal pathway which is activated in various cancers. Therefore, bisebromoamide (**17**) has potential as a lead for anticancer drugs.

**Figure:**
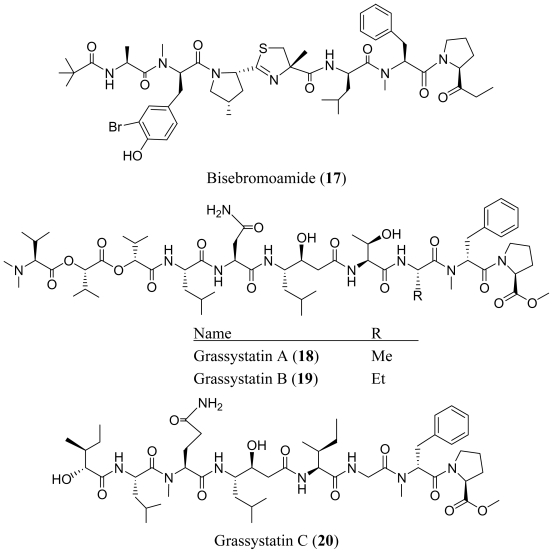


Three statine unit (γ-amino-β-hydroxyacid)-containing linear peptides, termed grassystatins A–C (**18**–**20**), have been isolated from the marine cyanobacteria *Lyngbya confervoides* collected off Grassy Key in Florida [20]. Grassystatins A and B (**18**–**19**) showed similar potency and selectivity against cathepsin D (IC_50_ values of 26.5 nM and 7.27 nM, respectively) and E (IC_50_ values of 886 pM and 354 pM, respectively). The increased affinity for cathepsin E over cathepsin D may be interpreted by the interaction of the polar asparagine residue in grassystatins A and B (**18**–**19**) with glutamine-303 in cathepsin E, which corresponds to the nonpolar residue methionine-307 in cathepsin D. Grassystatin A (**15**) was observed to reduce antigen presentation by dendritic cells, a process thought to rely on cathepsin E. The truncated peptide analog grassystatin C (**20**), which consists of two fewer residues than grassystatins A and B (**18**–**19**), was less potent against both cathepsins D and E, but still selective for cathepsin E. The selectivity of grassystatins A–C (**18**–**20**) for cathepsin E over D (20–38-fold) suggests that these natural products may be useful tools to probe cathepsin E function. In addition, grassystatins A–C (**18**–**20**) were effective inhibitors of the metalloprotease TACE (tumor necrosis factor α converting enzyme) with IC_50_s of 1.23 μM, 2.23 μM and 28.6 μM respectively.

## 3. Cyclic Peptides

An assay-based screening program for new neuroactive compounds from cyanobacteria led to the discovery of alotamide A (**21**) [21]. Alotamide A (**21**) is a cyclic depsipeptide featuring three contiguous peptidic residues linked by a polyunsaturated dihydroxyheptaketide which has not been found in any other natural product. Alotamide A (**21**) displays an unusual calcium influx activation profile in murine cerebrocortical neurons with an EC_50_ of 4.18 μM. Although the molecular target and mechanism for the bioactivity of alotamide A (**21**) is still unclear, alotamide A (**21**) will attract considerable attention to further study of this new type of cyanobacterial neurotoxin.

**Figure:**
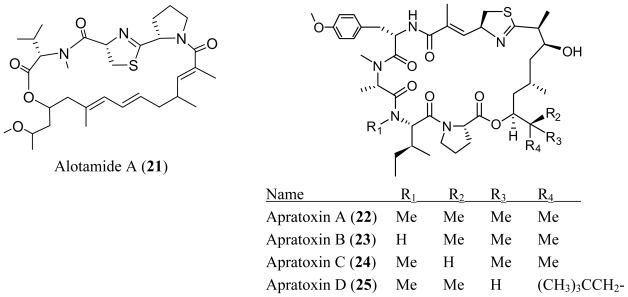


Apratoxin D (**25**) was isolated from a Papua New Guinea derived strains of the marine cyanobacteria *Lyngbya majuscula* and *Lyngbya sordida* [22]. Apratoxin D (**25**) contains the same macrocycle as apratoxins A–C (**22**–**24**) but possesses the unprecedented 3,7-dihydroxy-2,5,8,10,10-pentamethylundecanoic acid as the polyketide moiety. Apratoxin D (**25**) showed potent *in vitro* cytotoxicity against H-460 human lung cancer cells with an IC_50_ value of 2.6 nM, which is nearly equal in potency to that of apratoxin A (**22**). The similar cytotoxicity of apratoxin A (**22**) and apratoxin D (**25**) indicates that the cytotoxicity of apratoxins is not strongly impacted by the larger lipopeptide tail. The isolation of apratoxin D (**25**) from two distinct species of the genus *Lyngbya* supports the hypothesis of genetic transfer of natural product biosynthetic pathways between marine cyanobacteria [23].

A collection of the marine cyanobacterium *Lyngbya bouillonii* from Guam afforded the cytotoxic apratoxin E (**26**) [24]. Apratoxin E (**26**) displayed stronger cytotoxicity than its closest analog, semisynthetic *E*-dehydroapratoxin A (**27**) against several cancer cell lines derived from colon, cervix, and bone, ranging from 21 to 72 nM, yet is 5- to 15-fold less active than apratoxin A (**22**). It was speculated that the conformational alteration to apratoxin E (**26**), which results from the dehydration in the polyketide chain, reduces its activity.

**Figure:**
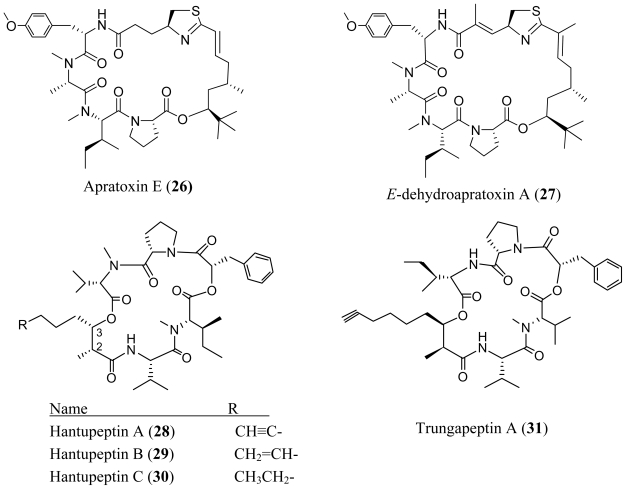


The marine *Lyngbya majuscula* from Pulau Hantu Besar, Singapore afforded a cyclodepsipeptide, hantupeptins A–C (**28**–**30**) [25,26]. Hantupeptin A (**28**), B (**29**) and C (**30**) all showed 100% brine shrimp mortality at 100 and 10 ppm. This was significantly higher than the activity reported for their closest analog trungapeptin A (**31**), which was only mildly toxic to brine shrimp [27]. Furthermore, *in vitro* cytotoxicity testing of hantupeptins A (**28**), B (**29**) and C (**30**) against the leukemia cell line MOLT-4, displayed IC_50_ values of 32 nM, 0.2 μM and 3.0 μM, respectively and 4.0 μM, 0.5 μM and 1.0 μM, respectively, against the breast cancer cell line MCF-7. In comparison, trungapeptin A (**31**), was reported to be inactive when tested at 10 μg/mL against KB or LoVo cells. It may be noteworthy to add that the hantupeptins have the 2*R*, 3*S* absolute configurations while the corresponding stereocenters in trungapeptin are 2*S*, 3*R.*

The marine *Lyngbya majuscula* collected from Palmyra Atoll produced palmyramide A (**32**), an unusual cyclic depsipeptide composed of three amino acids and three hydroxy acids [28]. The 2,2-dimethyl-3-hydroxyhexanoic acid unit (Dmhha) was also found in guineamide F (**33**) [29]. Palmyramide A (**32**) blocked the voltage gated sodium channel in neuro-2a cells (IC_50_ = 17.2 μM) and showed mild cytotoxicity against H-460 human lung carcinoma cells (IC_50_ = 39.7 μM). However, the cytotoxicity of guineamide F (**33**) was not reported. The planar structure of the 2,2-dimethyl-3-hydroxy-7-octynoic acid unit-containing dudawalamide A (**34**) was recently reported [30]. In an interesting correlation to the acyclic dragonamide A (**7**) and almiramides, it also exhibited anti-parasitic activity [31].

**Figure:**
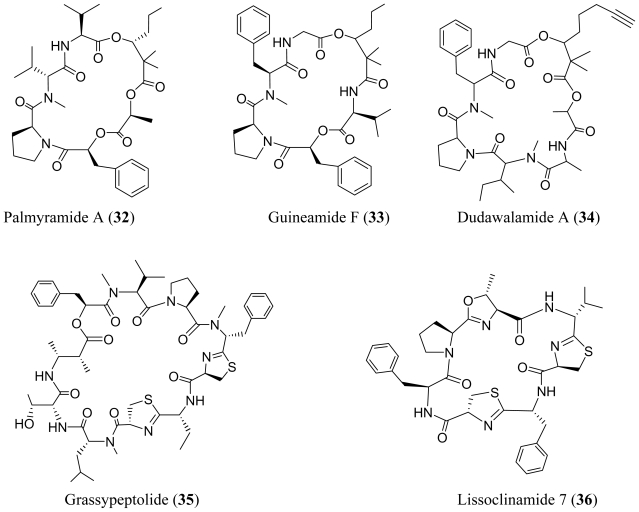


A collection of the cyanobacterium *Lyngbya confervoides* from the Florida Keys yielded grassypeptolide (**35**), a macrocyclic depsipeptide with an unusually high *D*-amino acid content, two thiazolines, and one *β*-amino acid [32]. Grassypeptolide (**35**) inhibited four cancer cell lines derived from human osteosarcoma (U2OS), cervical carcinoma (HeLa), colorectal adenocarcinoma (HT29), and neuroblastoma (IMR-32) with IC_50_ values from 1.0 to 4.2 μM. These data are within the range of IC_50_ values reported for lissoclinamide 7 (**36)** (53.7 nM to 21.5 μM), but in different cell lines [33,34]. Lissoclinamide 7 (**36**), the most cytotoxic of the lissoclinamide series, has two thiazoline rings with the same arrangement and stereoconfiguration as **35**. It has been shown that the thiazolines of **36** are important to its cytotoxic activity [34]. If this is the case, then it is very possible that grassypeptolide (**35**) and lissoclinamide 7 (**36**) share a similar mechanism of action.

A cyclodepsipeptide, termed carriebowmide (**37**) was isolated from the lipophilic EtOAc-MeOH-soluble fraction of *Lyngbya polychroa* collected from Carrie Bow Cay, Belize [35]. Carriebowmide (**37**) was also isolated from the same strain of *Lyngbya majuscula* that produced two depsipeptides, itralamides A and B (**53**–**54**) [41]. Carriebowmide (**37**) contains two rare amino acids, 3-amino-2-methylhexanoic acid and methionine sulfoxide. The lipophilic EtOAc-MeOH-soluble fraction from *Lyngbya polychroa* significantly deterred feeding by a natural assemblage of reef fish. This is presumably due to the presence of carriebowmide (**37**), however the effectiveness of the purified compound as a feeding deterrent was not determined.

**Figure:**
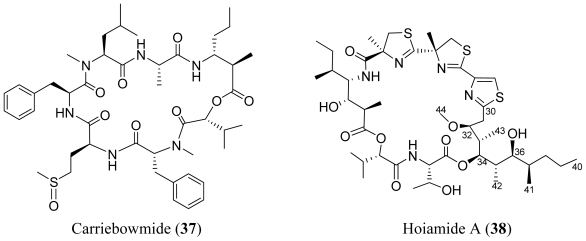


A combination of ^1^H NMR and bioassay-guided screening for neuroactive compounds from cyanobacteria led to the discovery of hoiamide A (**38**), a bioactive cyclic depsipeptide [36]. The 15 carbon subunit (C30 to C44) appears to be derived from a polyketide pathway. The pattern of oxidation and placement of pendant methyl groups suggests that the eleven-carbon main chain of Dmetua (5,7-dihydroxy-3-methoxy-4,6,8-trimethylundecanoic acid) may arise from the condensation of four propionates and one acetate unit. Hoiamide A (**38**) acted as a partial agonist at site 2 of the voltage-gate sodium channel α subunit, inhibited batrachotoxin-induced elevation of [Na^+^]_I_ in a concentration dependant manner, and exhibited modest cytotoxicity to cancer cells.

**Figure:**
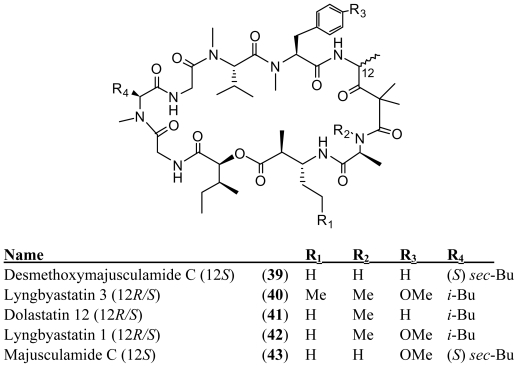


Desmethoxymajusculamide C (**39**) was isolated from *Lyngbya majuscula* collected in Fiji [37]. The closely related compounds, lyngbyastatin 3 (**40**), dolastatin 12 (**41**) and lyngbyastatin 1 (**42**), were determined to be mixtures of Ibu (4-amino-2,2-dimethyl-3-oxopentanoic acid units) epimers [*R* (major) and *S* (minor)] whereas the structurally related majusculamide C (**43**) is a single diastereomer with an *S*-Ibu unit. For **40**–**42**, but not **43**, significant NMR line broadening was observed. This was attributed to two factors: the presence of epimers and differing ratios of *cis/trans* conformers for individual epimers. Like majusculamide C (**43**) NMR line broadening was not observed for **39**, suggesting the presence of a single Ibu epimer. Desmethoxymajusculamide C (**39**) and its ring-opened form (generated by breaking the ester bond) demonstrated equivalent efficacy and solid tumor selectivity against four cell lines, including HCT-116, H-460, MDA-MB-435 and Neuro-2A. HCT-116 was the most sensitive cell line, with IC_50_ values of 20 and 16 nM, for **39** and its ring-open analog, respectively. This finding was discordant with the common viewpoint that the cyclic form is the bioactive form of the many peptides.

## 4. Cyclic Peptides with One or More Amide or Ester Branches

Tiglicamides A–C (**44**–**46**) were isolated from the Florida marine *Lyngbya confervoides* along with largamides A–C (**47**–**49**) [38,39]. The largamides and tiglicamides differ by only a single amino acid residue in the cyclic core. This single amino acid difference may result from assembly by an NRPS with adenylation domains having unusually relaxed specificity [40] or by a separate biosynthetic pathway. Both the largamides A–C (**47**–**49**) and the tiglicamides A–C (**44**–**46**) are serine protease inhibitors with selectivity for elastase over chymotrypsin and trypsin (Table 1). The carboxylic acid residue has little effect on the elastase inhibitory activity because the semi-synthetic largamide methyl esters **50**–**52** retained low-micromolar inhibitory activity.

**Figure:**
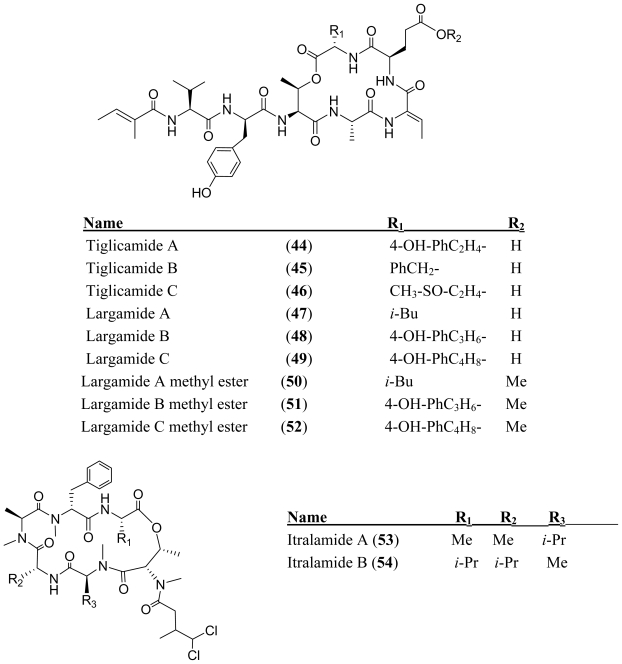


An eastern Caribbean collection of *Lyngbya majuscula* produced two depsipeptides, itralamides A and B (**53**–**54**) [41]. Itralamide B (**54**) displayed significant cytotoxicity in human embryonic kidney (HEK-293) cells (IC_50_ = 6 ± 1 μM). The closely related itralamide A (**53**) had a ten-fold lower potency than itralamide B (**54**). The difference in the cytotoxicity of itralamides A and B (**53**–**54**) demonstrates that biological activities can be dramatically altered by minor modifications of structure.

The investigation of the marine cyanobacterium *Lyngbya confervoides* collected from the southeastern coast of Florida led to the isolation of 3-amino-6-hydroxy-2-piperidone (Ahp) containing peptolide, pompanopeptin A (**55**), and a novel cyclic pentapeptide, pompanopeptin B (**56**) [42]. Pompanopeptin B (**56**) contains *N*-methyl-2-amino-6-(4′-hydroxyphenyl) hexanoic acid (*N*-Me-Ahpha) and is structurally related to carboxypeptidase-A inhibitors anabaenopeptins I (**57**) and J (**58**) which were isolated from the cyanobacterium *Aphanizomenon flos-aquae.* The *L*-Htyr and *N*-methyl-*L*-Ahpha, respectively in pompanopeptin B (**56**) were replaced by *L*-leucine/*L*-phenylalanine and *N*-methyl-*L*-alanine residues of **57** and **58** [43]. Pompanopeptin A (**55**) selectively inhibited trypsin over elastase and chymotrypsin, with an IC_50_ value of 2.4 μM (Table 1). The authors concluded that this selectivity is conferred by the arginine residue in the cyclic core. No activity data for **56** is reported.

**Figure:**
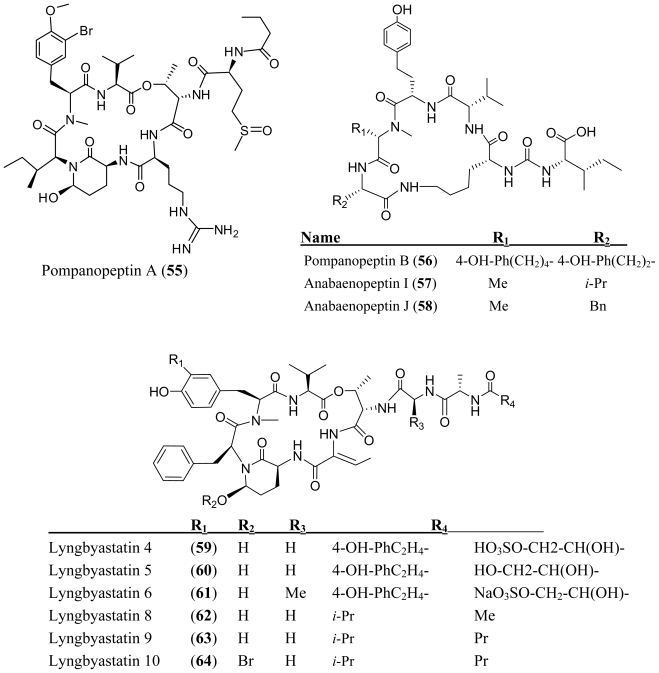


Several additional members of the Ahp (3-amino-6-hyroxy-piperidone) or Amp (3-amino-6-methoxy-piperidone) containing class of depsipeptides have been isolated and characterized from various species of *Lyngbya*. Lyngbyastatins 4–6 (**59**–**61**), were isolated from the marine cyanobacterium, *Lyngbya confervoides* which was collected off the Florida Atlantic coast [44,45]. Lyngbyastatins 8–10 (**62**–**64**) were isolated from the marine cyanobacterium *Lyngbya semiplena*, which was collected in Tumon Bay, Guam [46]. A *Lyngbya* sp., which was collected from a mangrove channel at Summerland Key in Florida, provided lyngbyastatin 7 (**65**) [45], somamide B (**66**) [47] and kempopeptins A and B (**67**–**68**) [48]. This class of compounds inhibits serine proteases, although the group exhibits a wide range of potency and varied selectivity (Table 1). When compared to the inhibitory activity of tiglicamides A–C (**44**–**46**), the Ahp containing depsipeptides are two to three orders of magnitudes more effective against porcine pancreatic elastase *in vitro* (Table 1). Like the tiglicamides (**44**–**46**) and the largamides (**47**–**49**), the Ahp containing serine proteases retain selectivity for elastase over chymostrysin or trypsin. Extensive structure activity studies as well as the crystal structures of the related desipeptide scyptolin A (**69**) bound to elastase [49,50] and cyanopeptolin A90720A bound to trypsin [51], revealed important interactions in the enzyme binding site and provided critical insights into the selectivity of this class of inhibitors. In the co-crystal structure of scyptolin-elastase, the threonine residue that forms ester bond occupies the S2 subsite of the protease. The valine which is C-terminal to this threonine binds the S1 subsite of the protease. The threonine and alanine of the pendant side-chain, bind subunits S3 and S4. The relative potencies of these new depsipeptides are consistent with earlier studies. Hydrophobic residues between the Thr that forms the ester bond of the depsipeptide and the Ahp residue impart selectivity for elastase as exemplified by the lynbyastatins (Abu), kempopeptin A (leucine) and scyptolin A (valine), whereas a basic residue at this position imparts selectivity for trypsin exemplified by kempopeptin B (lysine in **68**) and pompanopeptin A (argenine in **55**). In comparison with lynbyastatin 7 (**65**), lyngbyastatins 8–10 (**62**–**64**) exhibited weaker inhibition against porcine pancreatic elastase. Lyngbyastatins 8–10 (**62**–**64**) and lynbyastatin 7 (**65**) share the same depsipeptide core. Therefore, the reduced potency may be attributed to differences in the side chain residues. The exclusively hydrophobic residues in the pendant chain may form more favorable electrostatic interactions and hydrogen bonding with the enzyme. The fact that the protease-inhibitory activity is retained in the *O*-methylated (Amp) derivative, lyngbyastatin 6 (**60**), demonstrates that the hydroxyl group in the Ahp unit is not critical for the inhibition of elastase or chymotrypsin.

**Figure:**
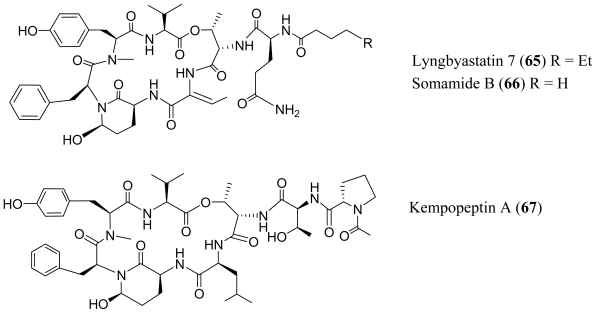


**Figure:**
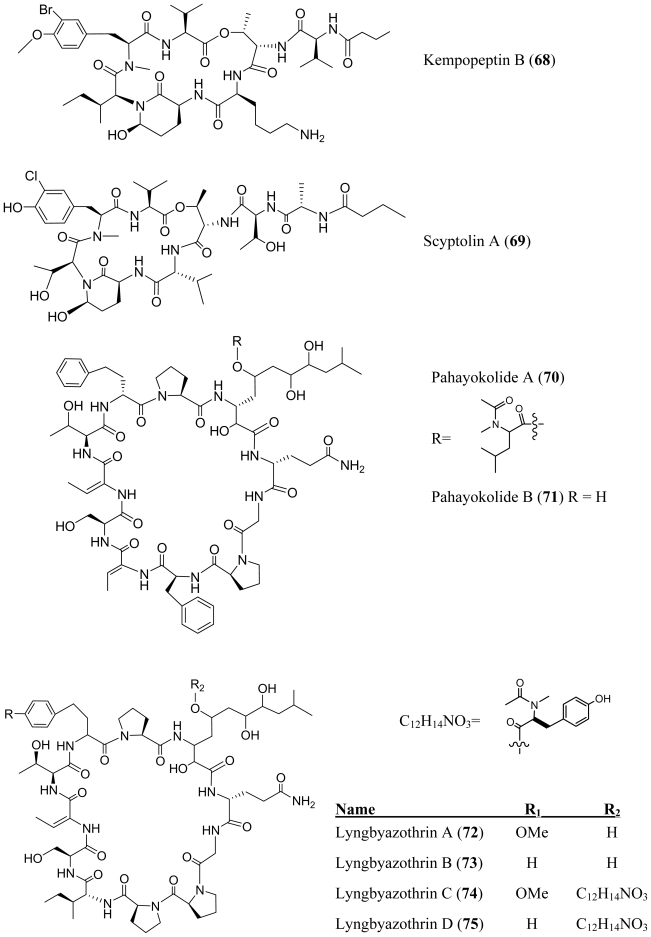


Pahayokolides A–B (**70**–**71**), lyngbyazothrins A–D (**72**–**75**) and schizotrin A (**76**) are remarkably similar in structure. Pahayokolides A–B (**70**–**71**) and lyngbyazothrins A–D (**72**–**75**) were produced by a freshwater *Lyngbya* sp. isolated from the Florida Everglades and the cultured *Lyngbya* sp. 36.91 SAG collected from Göttingen, Germany respectively [52–54]. On the other hand schizotrin A (**76**) and the tychonamides (**77**–**78**) were isolated from *Schizotrix* sp. [55] and *Tchyonema* sp. [56] respectively. Each of these cyclic undecapeptides contains the sequence (Val/Ile/Dhb)-Ser-Dhb-(Ser/Thr)-(homo-Phe/homo-Tyr)-Pro-X-Gln-Gly-Pro-(Pro/Phe), where X is an unusual, long-chain α,γ-hydroxy-β-amino acid. In the pahayokolides (**70**–**71**), the lyngbyazothrins (**72**–**75**) and schizotrin A (**76**) the α,γ-hydroxy-β-amino acid is 3-amino-2,5,7,8-tetrahydroxy-10-methylundecanoic acid (Athmu) and in tychonamides (**77**–**78**) it is a 3-amino-2,5,7,-trihydroxy-8-phenyloctanoic acid moiety (Atpoa). The γ-hydroxy group may or may not be decorated through an ester linkage to an *N*-acetyl-*N*-methyl leucine (pahayokolides and tychonamides) an *N*-butyroyl-*N*-methyl alanine (schizotrin A) or an *N*-acetyl-*N*-methyl tyrosine (lyngbyazothrins). To the best of our knowledge, these two long-chain α,γ-hydroxy-β-amino acids have not been observed elsewhere. It may be noteworthy that the pahayokolides, schizotrin, the lynbyazothrins and tychonamides, which are remarkably similar in structure, were all isolated from freshwater species. Pahayokolide A (**70**) inhibits a number of cancer cell lines over a range of concentrations (IC_50_ varied from 2.13 to 44.57 μM), is acutely toxic to zebrafish embryos (LC_50_ = 2.15 μM), and only marginally toxic against brine shrimp at the highest concentrations tested (1 mg/mL) [57]. The mixture of lyngbyazothrins A (**72**) and B (**73**) showed only low antimicrobial activity against *Micrococcus flavus*, whereas the mixture of lyngbyazothrins C (**74**) and D (**75**) was active against *Bacillus subtilis*, *Escherichia coli*, *Pseudomonas aeruginosa*, and *Serratia marcescens*. It seems that the acyl residue at C-5 of Athmu plays an important role in antimicrobial activity. This assumption was supported by the activity and structure of pahayokolides A–B (**70**–**71**).

**Figure:**
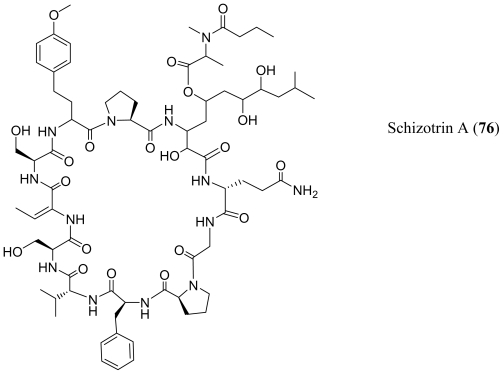


**Figure:**
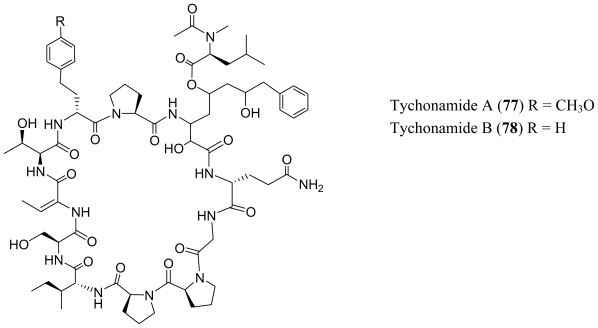


Fifty new peptides isolated from *Lyngbya* species, reported since 2006 have been reviewed and their bioactivities discussed. All but six of these new compounds were derived from marine species of *Lyngbya*, although it is likely that this represents a sampling bias rather than a significant difference in biosynthetic capability between marine and freshwater species. The genus *Lyngbya* appears to be an emerging source of bioactive peptides. A search of Chemical Abstracts for original research articles using the keywords “*Lyngbya”* and “peptide” returns 105 citations from 1980 to the present, of which nearly 1/3 have been published during the time covered by this review. In a recent review, Welker described seven major and three minor structural classes which encompass just over 50% of all known cyanaobacterial peptides [12]. In contrast, only 11 of the 50 new peptides covered in this review fall within one of the identified classes: pompanopeptin A (**55**), lyngbyastatins 4–10 (**59**–**64**) and kempopeptins A and B (**67**–**68**) belong to the larger group of cyanopeptolins. While pompanopeptin B (**56**) falls within the anabaenopeptins. *Lyngbya* derived peptides share many features with other cyanobacterial peptides, such as N-methylation, the incorporation of D-amino acids, thiazolines or oxazolines and subunits of polyketide or mixed-polyketide and non-ribosomal peptide origin. Nonetheless, the majority of the peptides covered is unique to *Lyngbya* species and encompass one or only a few analogs. It is common for cyanobacteria, to produce more than one member of a certain structural class. This phenomenon is partially a result of relaxed specificity of biosynthetic enzymes [58]. As prokaryotic polyketide synthases (PKS) can show considerable substrate tolerance [59], so may adenylation domains of non-ribosomal peptide synthetase (NRPS) accept different amino acids within a group of polar or nonpolar residues [60]. Natural structural diversification may also be the result of slight genetic variation or a slightly changed environment. With the possible exception of the linear dragonamides and almiramides, it is likely that these peptides are made non-ribosomally. To date, five biosynthetic pathways for *Lyngbya* peptides have been identified; barbamide, curacin A, lyngbyatoxin, jamacamide, and hectochlorin [61–65]. These pathways serve to illustrate the tremendous biosynthetic diversity offered by this genus.

A number of the reviewed *Lyngbya* peptides, especially acyclic peptides (**8**–**12** and **17**) and cyclic peptides without branches (**25**–**26**, **28**–**30**, **32**, **35**, **39**, **53**–**54**) exhibit cytotoxic activity against the various cancer cell lines. Only one cyclic *Lyngbya* peptide with branches, pahayokolide A inhibits a number of cancer cell lines over a range of concentrations. However, most of the reviewed cyclic branched-peptides (**44**–**49**, **55**, **59**–**65**, **67**–**68**) inhibit proteases with a wide range of potency and varied selectivity. Bioactive *Lyngbya* peptides may be considered to be a valuable pool of lead compounds in structure-based drug design and discovery.

## Figures and Tables

**Table 1:** Inhibition of serine proteases by peptides from the cyanobacterial genus *Lyngbya* (IC_50_, μM).

Name	Elastase	Chymotrypsin	Trypsin	Reference
Tiglicamide A (**44**)	2.14	>50	>50	[38]
Tiglicamide B (**45**)	6.99	>50	>50	[38]
Tiglicamide C (**46**)	7.28	>50	>50	[38]
Largamide A (**47**)	1.41	>50	>50	[39]
Largamide B (**48**)	0.53	>50	>50	[39]
Largamide C (**49**)	1.15	>50	>50	[39]
Pompanopeptin A (**55**)	2.4			[42]
Lyngbyastatin 4 (**59**)	0.0139 or 0.03	4.3 or 0.3	>30	[44,45]
Lyngbyastatin 5 (**60**)	0.0032	2.8	>30	[45]
Lyngbyastatin 6 (**61**)	0.0033	2.5	>30	[45]
Lyngbyastatin 8 (**62**)	0.0083 or 0.047	2.5	>30	[45,46]
Lyngbyastatin 9 (**63**)	0.123	/	/	[46]
Lyngbyastatin 10 (**64**)	0.210	/	/	[46]
Lyngbyastatin 7 (**65**)	0.120	/	/	[46]
Somamide B (**66**)	0.0095	4.2	>30	[47]
Kempopeptin A (**67**)	0.32	2.6	>67	[48]
Kempopeptin B (**68**)	>67	>67	8.4	[48]
Scyptolin A (**69**)	2.8	/	>446	[50]
